# Simulation Research on Impact Contact Behavior between Coal Gangue Particle and the Hydraulic Support: Contact Response Differences Induced by the Difference in Impacted Location and Impact Material

**DOI:** 10.3390/ma15113890

**Published:** 2022-05-30

**Authors:** Yang Yang, Yao Zhang, Qingliang Zeng, Lirong Wan, Qiang Zhang

**Affiliations:** 1 College of Mechanical and Electrical Engineering, Shandong University of Science and Technology, Qingdao 266590, China; sdkdzhangyao@126.com (Y.Z.); sdkdlirongwan@126.com (L.W.); sdkdzhangqiang@126.com (Q.Z.); 2 Shandong Provincial Key Laboratory of Mining Mechanical Engineering, Qingdao 266590, China; 3 College of Information Science and Engineering, Shandong Normal University, Jinan 250358, China

**Keywords:** coal and gangue, impact contact, response differences, equivalent stiffness, rigid–flexible coupling, different parts

## Abstract

In the process of top coal caving, coal gangue particles may impact on various parts of the hydraulic support. However, at present, the contact mechanism between coal gangue and hydraulic support is not entirely clear. Therefore, this paper first constructed the accurate mathematical model of the hydraulic cylinder equivalent spring stiffness forming by the equivalent series of different parts of emulsion and hydraulic cylinder, and then built the mesh model of the coal gangue particles and the support’s force transmission components; on this basis, the rigid–flexible coupling impact contact dynamic model between coal gangue and hydraulic support was established. After deducing contact parameters and setting impact mode, contact simulations were carried out for coal particles impacting at the different parts of the support and coal/gangue particles impacting at the same component of the support, and the contact response difference in the support induced by the difference in impacted component and coal/gangue properties was compared and studied. The results show that the number of collisions, contact force, velocity and acceleration of impacted part are different when the same single coal particle impact different parts of the support. Various contact responses during gangue impact are more than 40% larger than that of coal, and the difference ratio can even reach 190%.

## 1. Introduction

Top coal caving mining is the important mining method for thick and extra-thick coal seams: in the coal dropping stage of the top coal caving, the hydraulic support is parceled in the floor rock and coal gangue granule space body [1,2,3,4,5,6,7]. The large amount of particles and the continuous coal gangue dropping process will bring the impact contact of coal gangue and the hydraulic support. Due to the presence of flexible devices such as the hydraulic cylinder in the hydraulic support structure, the local impact contact behavior can cause the vibration of the whole hydraulic support. Grasp the impact contact characteristics and the contact response differences between coal gangue and the hydraulic support, is the foundation to study the interaction law between coal gangue or the surrounding rock and the hydraulic support and the precondition of coal gangue recognition in top coal caving. It is significant for the improvement of the hydraulic support application performance and advancement of top coal caving technology.

In the early stage, many studies have been carried out on the working characteristics of the hydraulic support and coal gangue recognition. Zhang et al. [8] studied the bearing characteristics of the hydraulic support by carrying out internal and external loading tests under different heel block contact conditions. Wang et al. [9] completed the stress and stability analysis of the prop by applying the load to the prop of the hydraulic support. Wang et al. [10] theoretically studied the coupling relationship of strength, stiffness and stability between the hydraulic support and surrounding rock. Ma et al. [11] studied the displacement and stress distribution of hydraulic support by stress testing and finite element analysis. Sun et al. [12] investigated load-bearing characters of hydraulic-powered roof support with dual telescopic legs. Xie et al. [13] studied the load-bearing characteristics of the top beam in a full range by constructing the spatial mechanical model of the top beam separation body of the four-column support. Wang et al. [14] established the plane mechanics model of four-column supporting shield support to study its adaptation. Ren et al. [15] designed the 1:2 reduced-scale hydraulic support to carry out the dynamic impact test and to study the response characteristics of hydraulic support under dynamic impact load. Gao et al. [16] added a step load to simulate the impact of the roof on the hydraulic support, so as to simulate and study the force transfer characteristics of the hydraulic support under the impact of roof pressure or coal lost gangue. Zhai et al. [17] used Amesim to establish the impact model of the column hydraulic system of the hydraulic support composed of the force principal model, safety valve model and weight model, then studied the impact response characteristics of the column hydraulic system. Luo et al. [18] studied the relationship between the impact force and the response speed of the hydraulic system by building the simulation model of the column hydraulic system of the hydraulic support. According to the existing research findings, one of the existing problems in the current study is that the interaction between coal gangue or the surrounding rock and the hydraulic support, or the working characteristics of the hydraulic support under dynamic load, are mainly studied by applying an equal load. The load is obtained through estimation, but the contact course between coal gangue or the surrounding rock and the support is ignored. The research scheme has a large error. The second problem existing in the current study is that there are few related researches on the interaction differences between coal gangue and the support. According to the Flores contact theory and the flexible energy absorption characteristics of the metal plate, Yang et al. [19,20] constructed the impact contact dynamics theoretical model between the single particle and the metal plate, the influence of impact velocity, recovery coefficient, material parameters, structure size, impact position and supporting spring stiffness on the dynamic contact response of the system was studied. However, the structural system is simple, and the study is not involved in the interaction between coal gangue and the multi-body combination hydraulic device such as the hydraulic support. Wan et al. [21,22] and Yang et al. [23] took the dynamic impact contact behavior of coal gangue and the tail beam as the research object, constructed the rigid–flexible coupling contact dynamic model of coal gangue impacting the tail beam structure and the dynamic contact finite element simulation model between the coal gangue particle and the tail beam under different constraints, respectively. The influence of impact angle, impact height, impact position, particle radius and particle material on dynamic contact response of the tail beam is studied. However, only the contact between the particle and the tail beam or equivalent structure of the tail beam is constructed, and the influence of the top beam, shield beam, front and rear connecting rod of the hydraulic support on the contact behaviors is not considered. Zeng et al. [24] established the numerical simulation model of the hydraulic support, considering the whole structure of the hydraulic support, but when studying the response of the hinge point under the condition of the single coal gangue particle impacting the hydraulic support, the contact behavior between the particles and the hydraulic support is imposed by the calculated contact force. At present, the contact response and response difference when the single coal gangue particle impact the different parts of the hydraulic support have not been studied in a reliable way. The third problem existing in current research is that some existing research carries out research on the hydraulic support by replacing the hydraulic cylinder with the equivalent spring damping module, but the spring stiffness model is not accurate. For example, Liang et al. [25,26] and Wan et al. [27] establish the equivalent stiffness model of the hydraulic cylinder just with the consideration of the hydraulic oil compressibility, Liu et al. [28] and Yang et al. [29] only consider the elasticity of the hydraulic oil and the cylinder body to establish elastic equivalent stiffness model of the hydraulic cylinder. In fact, the elasticity of the piston rod and other structures will also affect the equivalent stiffness of the hydraulic cylinder. The fourth problem existing in current research is that although there are many related studies in the field of coal gangue recognition, the selection of effective coal gangue recognition parameters and the research of selection basis are rarely involved. Dou et al. [30] identified coal and gangue with four kinds of working space by image analysis and Relief-SVM. Lai et al. [31] applied the multispectral technology and two-dimensional autoencoder in their research on coal gangue recognition. Liu et al. [32] carried on the Hilbert spectrum analysis to the vibration signal of the tail beam so as to study the coal gangue interface identification technology. Song et al. [33] collected the vibration signal and the sound signal, and then proposed an effective minimum enclosing ball (MEB) algorithm plus the support vector machine (SVM) to coal gangue rapid detection in top coal caving. Pu et al. [34] conducted coal gangue image identification by the convolutional neural network and transfer learning. Hou et al. [35] established coal gangue classification system based on the difference between surface texture and gray scale characteristics, and identified coal gangue by image feature extraction and artificial neural network. Zhang et al. [36] analyzed the distribution characteristics of natural gamma rays in roof coal and rock, established the relationship between radiation intensity and coal gangue content, and identified mixed coal and gangue by radiation signal detection. Alfarzaeai et al. [37] studied coal gangue identification by convolutional neural networks and thermal images. Zhang et al. [38] used infrared imager with low emissivity to improve the coal gangue recognition accuracy based on liquid intervention. Yang et al. [39] used vibration, sound, pressure and other signals to classify and identify the mixing ratio of coal and gangue mixture. Yan et al. [40] used multi-spectral imaging technology and YOLOv5.1 target detection method to conduct the intelligent recognition and classification of coal and gangue. Yuan et al. [41] used six different classification methods to classify and identify coal and gangue by constructing the sample library of top coal caving sound signals. Wang et al. [42] constructed the lightweight accurate and fast recognition model of gangue rate, developed the intelligent image acquisition system and enhanced dust removal algorithm, and realized the image identification of coal gangue in the process of coal discharging. These studies directly applied the above methods in coal gangue recognition, but did not clarify the root causes or gist of recognition parameters or method selection, and did not analyze and discuss coal gangue identifiability at the theoretical level.

In top coal caving, the large number of coal gangue particles and the distribution characteristics of drawing space lead to the contact between coal gangue and various parts of the hydraulic support. Contact behavior between coal gangue particles and the hydraulic support involves the evolution of contact state and the real-time transmission of force. Overall, due to the lack of research method that can accurately describe the whole contact process between particles and hydraulic support, the precise contact characteristics and contact difference characteristics between coal gangue and the hydraulic support are not completely clear, in particular, the contact characteristics and contact difference between coal gangue and the different parts of the hydraulic support have not been studied. As a result, the selection of coal gangue recognition media lacks the theoretical basis in the research of coal gangue recognition technology. In view of the existing problems and deficiencies in the present studies and in order to further clarify the contact response characteristics and the contact response differences law between coal gangue and the hydraulic support, this paper proposed the idea of quantitative research, and the impact contact behavior between single particle coal gangue and the hydraulic support are taken as the research target. A rigid–flexible coupling impact contact simulation model between coal gangue and the hydraulic support is proposed to study the system dynamic response. For this purpose, an accurate model of the hydraulic cylinder liquid stiffness (equivalent spring stiffness) series by five different parts stiffness is firstly established. The rigid–flexible coupling impact contact simulation model between the coal gangue particle and the hydraulic support is established by combining the mesh model of the particle and the main force transmission components of the hydraulic support as well as the multi-body rigid dynamics simulation model of the hydraulic support. On the basis of determining the contact parameters and impact modes, the contact dynamics simulation analysis will be carried out when coal gangue impacts the different components of the hydraulic support with the same height and when the same size coal/gangue impacts the same position of the hydraulic support, respectively. Impact contact response characteristics when coal gangue impacts the different parts of the hydraulic support will be studied. Through comparative analysis, the difference rule of system impact contact responses caused by the difference in the impacted component and the particle material property will be determined, so as to explore the theoretical basis for the selection of coal gangue recognition media.

Our contributions in this paper are fourfold.

(1) Considering the compression elasticity of the emulsified liquid, piston rod, tail of the piston rod, bottom of the cylinder and the circumferential extension stiffness of the cylinder, a more accurate equivalent spring stiffness mathematical model of the hydraulic cylinder is established.
(2) The traditional scheme that studied the interaction between coal rock and the hydraulic support by replacing the contact process between coal gangue and the hydraulic support with force load is cancelled. The quantitative research method is put forward. Through the establishment of the rigid–flexible coupling impact contact dynamics simulation model between coal gangue and the hydraulic support, the contact response between particles and the hydraulic support is studied, which provides the direct and effective research method for the interaction between coal gangue or surrounding rock and the hydraulic support.
(3) Through the contact characteristics analysis between coal particles and the different parts of the hydraulic support, the variation rule of the system contact response caused by the change in the impact position is obtained.
(4) Through the contact response study of the direct contact parts, indirect related parts and the parts connection units after coal gangue impact, the contact difference characteristics between coal gangue and the hydraulic support are clarified, and the available parameters for coal gangue recognition are determined accordingly.

## 2. Establishment of the Hydraulic Cylinder Equivalent Stiffness Mathematical Model

In the hydraulic system of the hydraulic support, the props and the tail beam jack (collectively referred to as the hydraulic cylinder) are solid–liquid coupling-compressible devices of the steel structure and high pressure emulsion, and the hydraulic oil in the hydraulic cylinder cavity has the compressibility. According to previous studies, when dynamic software is used to analyze the dynamic characteristics of the hydraulic support, the equivalent spring damping module is usually used to replace the hydraulic cylinder.

**Figure 1:**
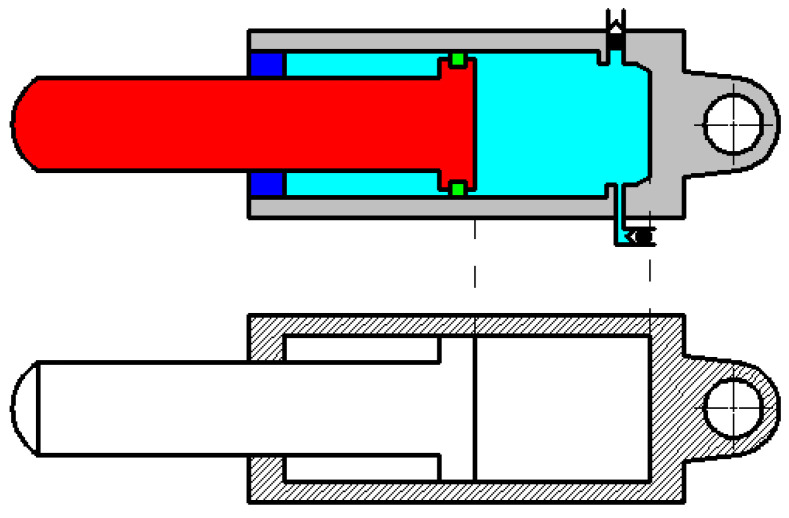
Stiffness calculation model of the hydraulic cylinder.

In order to obtain accurate impact contact dynamic response between coal gangue and the hydraulic support, the equivalent stiffness of the hydraulic cylinder in the working process should be determined accurately first when using spring damping to analyze the dynamic characteristics of the hydraulic support. The prop and the tail beam jack in this paper are all single telescopic hydraulic cylinder. Taking the tail beam jack as the example, as shown in Figure 1, observation shows that when bearing the external load, not only the hydraulic oil in the hydraulic cylinder and part of the cylinder body contacted with oil are stressed, but also the piston rod, the tail of the piston rod and the bottom of the hydraulic cylinder are stressed. High pressure emulsion is filled between the piston rod and the prop cylinder body; the piston rod, the internal oil liquid and the cylinder body are regarded as the spring respectively; each part of springs are in series with each other. The equivalent spring stiffness of the hydraulic cylinder actually consists of the equivalent compression stiffness of the emulsified liquid, the equivalent compression stiffness of the piston rod, the equivalent compression stiffness of piston rod tail, the circumferential extension stiffness of the hydraulic cylinder and the axial compressive stiffness of the hydraulic cylinder bottom. The stiffness coefficient of each part is as follows:

Equivalent compression stiffness of the emulsified liquid:

(1) $$\left\{ \begin{array}{l} K_{Y}=\frac{\Delta F_{H}}{\Delta l}=\frac{\Delta p\cdot S_{H}}{\Delta l}\\ E_{r}=\frac{\Delta p\cdot V_{0}}{\Delta V}\\ V_{0}=l\cdot S_{H}\\ \Delta V=\Delta l\cdot S_{H}\\ S_{H}=\frac{\pi d^{2}}{4} \end{array}\right.$$

(2) $$K_{Y}=\frac{E_{r}\cdot S_{H}}{l}=\frac{\pi d^{2}}{4}\cdot \frac{E_{r}}{l}$$

where $\Delta F_{H}$ is the variation of the hydraulic oil pressure, $S_{H}$ is the liquid column cross-sectional area, Δp is the variation of the pressure intensity of the hydraulic oil, $E_{r}$ is the volume elastic modulus of the emulsion liquid, l is the height of the emulsion liquid column, d is the inside diameter of the hydraulic cylinder and the diameter of the emulsion liquid column.

Circumferential extension stiffness of the cylinder body:

(3) $$\left\{ \begin{array}{l} \int_{0}^{\pi }\Delta p\cdot \frac{d}{2}\cdot l\cdot \sin\theta_{Z}d\theta_{Z}=2\sigma_{Z}\cdot l\cdot \frac{\left(D-d\right)}{2}\\ \sigma_{Z}=E_{GT}\cdot \varepsilon_{GT}\\ \varepsilon_{GT}=\frac{\Delta d/2}{d/2}\\ K_{GZ}=\frac{\Delta F_{Z}}{\Delta d}=\frac{\Delta p\cdot \pi \cdot d\cdot l}{\Delta d} \end{array}\right.$$

(4) $$K_{GZ}=\frac{\pi \cdot l\cdot (D_{d}-d)\cdot E_{GT}}{d}$$

where $D_{d}$ is the outside diameter of the cylinder body, $\sigma_{Z}$ is the circumferential stress of the cylinder body, $\varepsilon_{GT}$ is the circumferential strain of the cylinder body, $\theta_{Z}$ is the circumferential pressure angle, $E_{GT}$ is the elastic modulus of the cylinder body, Δd is the inner diameter variation of the hydraulic cylinder body, $\Delta F_{Z}$ is the variation of the circumferential pressure in the inner wall of the cylinder body.

Axial compressive stiffness of the cylinder bottom:

(5) $$\left\{ \begin{array}{l} K_{GD}=\frac{\Delta F_{GD}}{\Delta l_{GD}}\\ \sigma_{GD}=E_{GD}\cdot \varepsilon_{GDH}\\ \varepsilon_{GDH}=\frac{\Delta l_{GD}}{l_{GD}}\\ S_{H}\cdot \sigma_{GD}=\Delta F_{GD}\\ S_{H}=\frac{\pi d^{2}}{4} \end{array}\right.$$

(6) $$K_{GD}=\frac{\pi d^{2}}{4}\cdot \frac{E_{GD}}{l_{GD}}$$

where $l_{GD}$ is the length of the hydraulic cylinder bottom, $E_{GD}$ is the elastic modulus of the hydraulic cylinder bottom, $\varepsilon_{GDH}$ is the axial strain of the hydraulic cylinder bottom, $\sigma_{GD}$ is the axial stress of the hydraulic cylinder bottom, $\Delta l_{GD}$ is the axial elongation of $l_{GD}$.

Equivalent compression stiffness of the piston rod:

(7) $$\left\{ \begin{array}{l} K_{GG}=\frac{\Delta F_{GG}}{\Delta l_{GG}}\\ \sigma_{GG}=E_{GG}\cdot \varepsilon_{GGH}\\ \varepsilon_{GGH}=\frac{\Delta l_{GG}}{l_{GG}}\\ S_{GG}\cdot \sigma_{GG}=\Delta F_{GG}\\ S_{GG}=\frac{\pi {d_{1}}^{2}}{4} \end{array}\right.$$

(8) $$K_{GG}=\frac{E\cdot S_{GG}}{l_{GG}}=\frac{\pi {d_{1}}^{2}}{4}\cdot \frac{E_{GG}}{l_{GG}}$$

where $l_{GG}$ is the length of the piston rod, $E_{GG}$ is the elastic modulus of the piston rod, $d_{1}$ is the diameter of the piston rod, $\Delta l_{GG}$ is the elongation of the piston rod, $\sigma_{GG}$ is the axial stress of the piston rod, $\varepsilon_{GGH}$ is the axial strain of the piston rod, $S_{GG}$ is the cross-sectional area of the piston rod.

Equivalent compression stiffness of the piston rod tail:

(9) $$\left\{ \begin{array}{l} K_{GGW}=\frac{\Delta F_{H}}{\Delta l_{GGW}}\\ \sigma_{GGW}=E_{GGW}\cdot \varepsilon_{GGWH}\\ \varepsilon_{GGWH}=\frac{\Delta l_{GGW}}{l_{GGW}}\\ S_{H}\cdot \sigma_{GGW}=\Delta F_{H}\\ S_{H}=\frac{\pi d^{2}}{4} \end{array}\right.$$

(10) $$K_{GGW}=\frac{\pi d^{2}}{4}\cdot \frac{E_{GGW}}{l_{GGW}}$$

where $l_{GGW}$ is the length of the piston rod tail, $E_{GGW}$ is the elastic modulus of the piston rod tail, $\varepsilon_{GGWH}$ is the axial strain of the piston rod tail, $\sigma_{GGW}$ is the axial stress of the piston rod tail, $\Delta l_{GGW}$ is the axial elongation of $l_{GGW}$.

After each part is connected in series, the equivalent spring stiffness of the hydraulic cylinder can be obtained as follows:

(11) $$\begin{array}{l} K_{Q}=1/(\frac{1}{K_{Y}}+\frac{1}{K_{GZ}}+\frac{1}{K_{GD}}+\frac{1}{K_{GG}}+\frac{1}{K_{GGW}})\\ =\frac{K_{Y}K_{GZ}K_{GD}K_{GG}K_{GGW}}{K_{GZ}K_{GD}K_{GG}K_{GGW}+K_{Y}K_{GD}K_{GG}K_{GGW}+K_{Y}K_{GZ}K_{GG}K_{GGW}+K_{Y}K_{GZ}K_{GD}K_{GGW}+K_{Y}K_{GZ}K_{GD}K_{GG}} \end{array}$$

## 3. Construction of the Rigid–Flexible Coupling Impact Contact Dynamic Model between Coal Gangue and the Hydraulic Support

The purpose of this paper is to determine the contact response differences between coal gangue and the hydraulic support through the impact contact behavior analysis of coal gangue and the hydraulic support, and to reveal the impact contact characteristics and response difference law when coal gangue impacts the different parts of the support. Due to the large number and complex shape of the underground coal gangue particles as well as the existence of anisotropy, direct theoretical or simulation modeling is difficult to achieve. Moreover, the contact position of irregular shaped particles will cause the change in the equivalent contact radius and the contact responses, so it is impossible to study the influence parameters and the changing law of the contact characteristics qualitatively by using irregular shape. In order to conduct a qualitative study and reveal coal gangue contact difference characteristics caused by their own attribute differences, this paper conducted regular treatment to coal and gangue, uniformly treating coal and gangue particles as spheres, ignoring the plastic deformation and brittle damage of particles, and ignoring the influence of rock micro-cracks [43] on contact response. For quantitative analysis, only the impact behavior of single coal gangue particles and the hydraulic support was studied.

**Figure 2:**
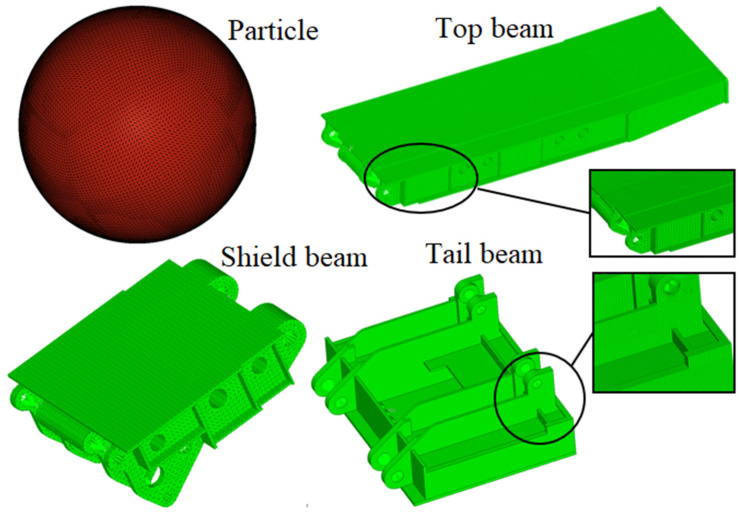
Mesh generation of sphere and the main parts of the hydraulic support.

In order to improve the accuracy of simulation results, the rigid–flexible coupling impact contact dynamic model between coal gangue and the hydraulic support is established to study the impact behavior. Particles and the main impacted part of the top coal caving hydraulic support such as top beam, the shield beam and the tail beam are mesh first, the structure after grid division is shown in Figure 2. Due to the movement and force transfer of the connection area of each component in the working process, so the grids of the connection areas such as the column nest surface of the top beam and the base and the pinhole inner surface of the other components are defined as rigid. In order to ensure the effect of force transmission, the front and rear connecting rods are defined as rigid bodies.

**Figure 3:**
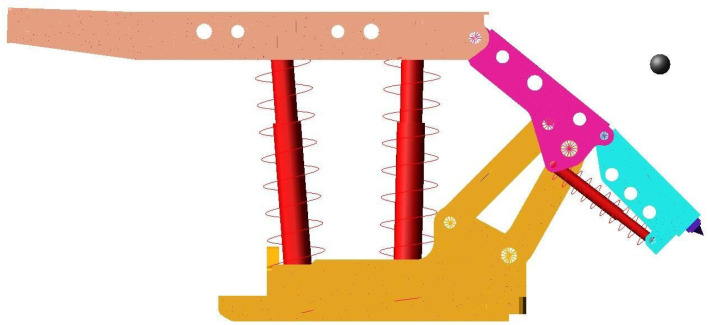
Dynamic model when coal gangue impacting the hydraulic support.

After the 3D model of coal gangue particles and top coal caving hydraulic support is introduced into Adams, the meshing particles, top beam, shield beam and tail beam files are introduced, respectively, into Adams to replace the corresponding solid file. The rigid area of the pin hole in the top beam, the shield beam, the tail beam, the front and rear connecting rods and the base is connected by the revolute pair. The props and the tail beam jack are equivalent replaced by spring damping modules. The rigid insert plate and the insert plate jack are fixed on the tail beam, and the rigid base is fixed in the space coordinate system. The impact position of coal gangue particles can be adjusted according to requirements, so as to realize the impact of coal gangue on different parts or positions of the support. The completed rigid–flexible impact dynamic model of coal gangue and the hydraulic support is shown in Figure 3.

## 4. Simulation of the Impact Contact Characteristics between Coal Gangue and Different Parts of the Hydraulic Support

### 4.1. Setting of the Contact Parameters

The radius of coal gangue particles is 2.5 × 10^−2^ m. The contact stiffness between coal gangue and the hydraulic support can be calculated by the contact stiffness calculation formula [44,45,46,47,48,49]:

(12) $$K=\frac{4\sqrt{R}}{3}\cdot E$$

Equivalent elastic modulus $\frac{1}{E}=\frac{1-\mu_{1}^{2}}{E_{1}}+\frac{1-\mu_{2}^{2}}{E_{2}}$, equivalent contact radius $\frac{1}{R}=\frac{1}{R_{1}}+\frac{1}{R_{2}}$, $E_{1}$, $\mu_{1}$, $E_{2}$ and $\mu_{2}$ are the elastic modulus and Poisson’s ratio of the two contact body, respectively, $R_{1}$ and $R_{2}$ are the contact radius of the contact position on two contact bodies, respectively. For the contact between the spherical particle and the surface of the hydraulic support, the surface can be equivalent to a spherical particle with infinite radius, i.e., $R_{2}\to \infty$, then the equivalent contact radius is $R=R_{1}$.

The props and the tail beam jacks are the single telescopic hydraulic cylinder, and the liquid column height in each cylinder is associated with the position of the hydraulic support. When the working height of the hydraulic support is 2.5 m and the coal dropping angle of the tail beam is 45°, the sizes of the props and the tail beam jack are shown in Table 1, respectively. The equivalent stiffness of the front row prop, back row prop and the tail beam jack are calculated as 9.5 × 10^7^ N/m, 9.6 × 10^7^ N/m and 1.1 × 10^8^ N/m according to Equation (11).

### 4.2. Contact Model in the Simulation

The essence of the impact contact behavior between the coal gangue particle and the hydraulic support is the nonlinear contact between the particle and the metal plate plane, so the contact model based on nonlinear spring damping in Adams is applied to its definition, the normal contact force can be combined describing by the Hertz contact theory-based elastic contact force and the system damping dissipative force, as shown in Equation (13) [50,51,52]:

(13) $$F_{n}=\left\{ \begin{array}{l} K\cdot \delta^{n}+STEP(\delta ,0,0,\lambda_{\max},c_{\max})\cdot \overset{\cdot }{\delta },\delta >0\\ 0,\delta \leq 0 \end{array}\right.$$

where δ is the relative deformation (particle compression), $\lambda_{\max}$ is the a positive real value specifying the boundary penetration to apply the maximum damping coefficient $c_{\max}$, $\overset{\cdot }{\delta }$ is the relative velocities of two bodies in contact, n is the nonlinear exponent of Hertz contact force.

The step function describing the system damping dissipative force in Adams can be further described as follows:

(14) $$STEP(\delta ,0,0,\lambda_{\max},c_{\max})=\left\{ \begin{array}{l} 0,\delta \leq 0\\ c_{\max}{\left(\frac{\delta }{\lambda_{\max}}\right)}^{2}\left(3-\frac{2\delta }{\lambda_{\max}}\right),0<\delta <\lambda_{\max}\\ c_{\max},\delta \geq \lambda_{\max} \end{array}\right.$$

### 4.3. Impact Mode of Particles and Different Parts of and Hydraulic Support

**Figure 4:**
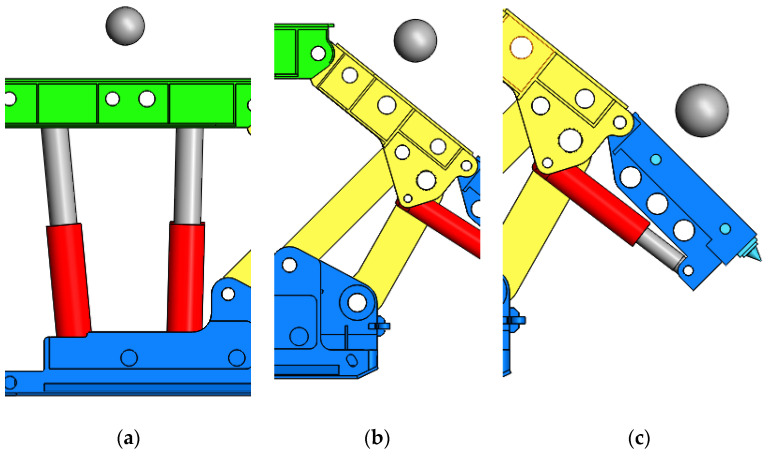
(**a**) Impact top beam; (**b**) Impact shield beam; (**c**) Impact tail beam; Impact modes of the particles and the different parts of the hydraulic support.

The impact position of coal gangue on the top beam was located at the axial center line of the top beam, as shown in Figure 4a. The center of the spherical particle was aligned with the axial midpoint of the front and rear props. In the process of coal gangue dropping, the particles collide frequently with the hydraulic support; however, restricted by the coal gangue dropping space, relative impact velocity between particles and the support is limited. The falling height of the particle free falling and impacting the top beam is defined as 0.8 m (the vertical distance between the center of the sphere and the top surface of the top beam); in this way, the impact contact velocity between particles and the support is limited. After the main structure is divided into grids and the jack is replaced by the spring damping module, the support under the action of gravity will generate structural deformation and vibration self-stability. When the particles fall from a height of 0.8 m and impact on the top beam, the support has not been self-stabilized when the particles contact with the top surface of the top beam, and the residual vibration cannot be ignored. We use “deceleration + free falling” compound movements to realize coal gangue low-velocity impact simulation with the hydraulic support, namely, to extend the fall time and weaken the influence degree of the system vibration on the simulation results by changing the initial relative position and applying an upward initial velocity. Here, we use the initial speed of 2.94 m/s, which is increased by 0.3 s for self-stable equilibrium time at the support.

When coal gangue impacts the shield beam, the center of the spherical particle and the midpoint of the axial centerline of the shield beam are located on the same vertical line, and the distance between the center of the spherical particle and the midpoint of the axial centerline of the shield beam is 0.8 m. The relative positions of the coal gangue and shield beam are shown in Figure 4b. The dip angle of the shield beam will lead to the possibility of collision contact between particles and the tail beam after the multiple collisions and separation between the particles and the shield beam, so contact is added between the particles and the shield beam as well as between the particles and the tail beam. When the coal gangue impacts the tail beam, the center of the spherical particle and the midpoint of the axial center line of the tail beam are located on the same vertical line, and the center of the spherical particle and the midpoint of the axial center line of the tail beam is 0.8 m. The relative positions of the coal gangue and tail beam are shown in Figure 4c.

### 4.4. Impact Contact Response Analysis between Coal Particles and the Different Parts of the Hydraulic Support

**Figure 5:**
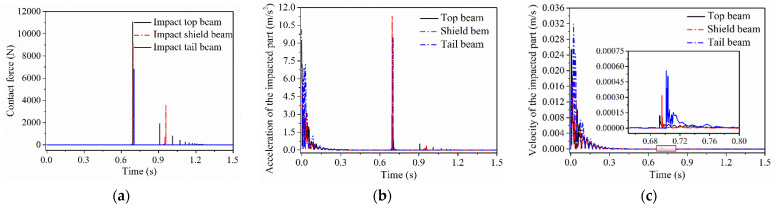
(**a**) Contact force; (**b**) Centroid acceleration; (**c**) Centroid velocity; Impact contact response of the different parts of the hydraulic support.

The impact behavior of particles and the different parts of the hydraulic support will lead to the change in the contact state and the force transfer effect, and further lead to a change in the contact response law. When coal particles free fall from the height of 0.8 m and impact on the designated positions of the top beam, the shield beam and the tail beam, the impact contact response of the different parts of the hydraulic support can be obtained, as shown in Figure 5.

The props and tail beam jacks are replaced by equivalent springs. In the process of particle free falling, the hydraulic support produces vibrated self-stable equilibrium under the combined action of gravity and the equivalent spring. According to the acceleration and velocity curve of the center of mass of the impinged part of the hydraulic support in Figure 5, the utmost velocity of the center of mass of the tail beam reaches the maximum of the three in the process of self-stable equilibrium, the utmost velocity of the center of mass of the shield beam is the minimum, while the utmost acceleration of the center of mass of the shield beam is the maximum, and that of the center of mass of the tail beam is the minimum. The collision and contact process between the particles and the support is very short and the contact force generated during the process is very large. The impact contact force curve between coal and the different positions of the hydraulic support shows that continuous collision between the coal particle and the top beam happened more than 10 times, and coal particles impacted with the shield beam twice and then separated, eventually coming into contact with the tail beam with rebound impact, but the particle just impacted with the tail beam once and then separated. The collision frequency mainly depends on the support structure, posture and the length of the upper surface metal plate on the various parts and the residual collision velocity of the particle.

When the particles collide with the top beam, the shield beam and the tail beam, these three parts have reached self-stable equilibrium. After self-stabilization, the top beam only deflected at a minimal angle, and the impact between the particles and the top beam was approximately vertical. Therefore, the utmost contact force between coal particles and the top beam was the largest during the initial impact contact. The deflection angle of the tail beam is greater than that of the shield beam, which is supported by the combined “rigid” structure of the top beam, front and rear connecting rods, while one end of the tail beam is in the flexible state of equivalent spring support, so the utmost contact force between particle and the shield beam is greater than that between particle and the tail beam, i.e., *F_top beam_ > F_shield bea_ > F_tail beam_*. Under the contact force, the relation of the maximum velocity and acceleration of the center of mass between the impinged parts is *v_top beam_* < *v_shield beam_ < v_tail beam_, a_top beam_ < a_tail beam_ < a_shield beam_*. During the collision between the particles and the hydraulic support, the system energy is greatly consumed, leading to a small degree of the system response in the second collision. Compared with the first collision, the contact force and the acceleration of the center of mass of the impacted parts in the second collision between coal particles and the top beam and the shield beam are reduced by more than 82%.

## 5. Study on the Impact Contact Response and Response Differences between Coal Gangue and the Hydraulic Support

Hydraulic support is the multi-body parallel equipment, and there are interactions between the different parts. The difference in the properties of coal and gangue determines the difference in the impact contact response between coal gangue and the metal plate of the hydraulic support, which will lead to the difference in the impact contact response between coal gangue and the main parts of the hydraulic support, and lead to the difference in the contact response between the associated parts and the parts’ joints. This section will determine the difference rule of the impact contact response between the coal gangue and the hydraulic support through the contact response analysis of the direct contact parts, indirect associated parts and parts connection units after coal gangue impact.

### 5.1. Difference in Contact Response of the Direct Contact Parts

**Figure 6:**
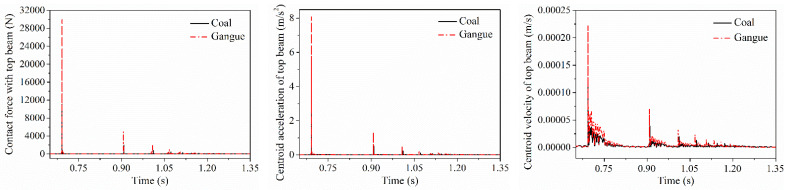
Impact contact response difference in the top beam.

**Figure 7:**
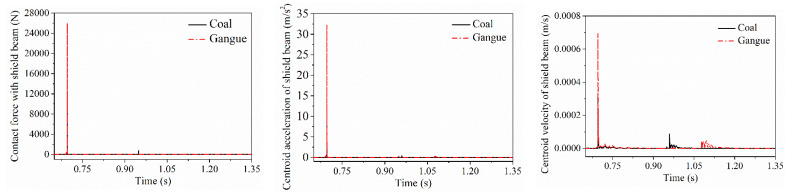
Impact contact response difference in the shield beam.

**Figure 8:**
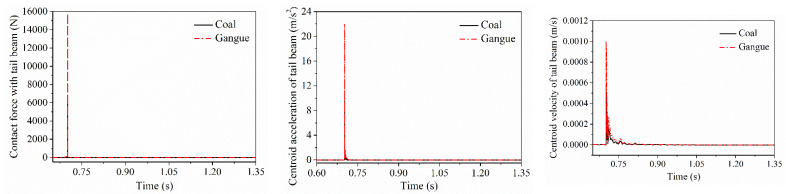
Impact contact response difference in the tail beam.

When coal gangue impacts on the top beam, the shield beam and the tail beam, respectively, the contact force between coal gangue and the direct contact parts as well as the acceleration and velocity changing curves of the direct impact parts are shown in Figure 6, Figure 7 and Figure 8. It can be seen that each impact contact process between coal gangue and the different positions of the hydraulic support is completed within a very short period of time. The contact force generated by the coal particle impacting is smaller than the gangue, and the vibration acceleration and velocity of the center of mass acquired by the impacted parts after coal particle impacting is also lower than the gangue. When colliding with the shield beam at the axial midpoint of the shield beam at the same height, the coal particle separated from the shield beam after two collisions and finally impacted on the tail beam, while the gangue separated from the shield beam after only once collision with the shield beam and then came into contact with the tail beam, that is, the impact frequency of coal gangue when impacting the shield beam was different.

**Figure 9:**
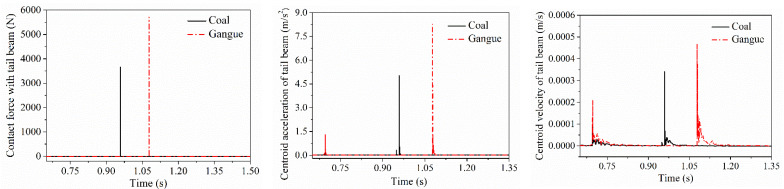
Impact contact response difference in the tail beam when the shield beam is impact directly.

After coal gangue impacted on the axial midpoint of the shield beam and separated with it, the impact contact force when the particle impact on the tail beam, velocity and acceleration curve of the tail beam are shown in Figure 9, respectively. The impact contact time between the gangue and the tail beam lags behind that of coal, but the contact force and the amplitude of the vibration velocity and acceleration of the tail beam centroid produced by the impact contact action were greater than that of coal.

To further clarify the difference in contact response of the direct contacted parts caused by the impact of coal gangue, the extreme values of each contact response in the process of initial collision contact were extracted, respectively, as shown in Table 2.

During the impact with the top beam, the shield beam and the tail beam, the maximum difference in the contact force generated by the impact between coal gangue particles and the top beam is the greatest, the maximum difference in the vibration acceleration of the impacted parts generated by the impact of the shield beam is the greatest, and the maximum difference in the vibration velocity of the impacted parts generated by the tail beam is the greatest. The contact forces generated by the impact of gangue are all more than 1.3 times higher than those generated by the impact of coal. The vibration velocity and acceleration of the center of mass obtained by the impinged parts after gangue impact is 0.8 times and 1.3 times higher than that of the impinged parts caused by coal impact, respectively. It can be seen that the contact response caused by the coal gangue when impact with the hydraulic support is obviously different, so it is feasible to identify coal and gangue based on the impact contact response. By comparing the difference ratio of coal gangue impact contact response, the difference ratio of the contact force, the vibration velocity and acceleration acquired by the centroid of the shield beam when coal gangue impacts on the shield beam are all the largest, reaching up to 1.9 times, 1.2 times and more than 1.9 times higher, respectively. The difference in the impacted parts contact response caused by the impact of coal gangue when impact on the tail beam is the smallest. Among the contact force, the centroid velocity and acceleration of the impinged parts, the difference ratio between the centroid acceleration of the impinged part and the contact force after coal gangue impact is similar, which is much higher than that of the centroid velocity of the impinged part.

After coal gangue impacted the shield beam and separated with it, the ultimate responses when the coal gangue final impacts on the tail beam such as the contact force, the vibration velocity and acceleration of the center of mass of the tail beam is shown in Table 3. Among them, the ultimate contact force was reduced by more than 59% compared with the contact force when impacting on the shield beam. The difference ratio of the contact force after coal gangue impact can reach above 0.6, the maximum difference ratio of the centroid acceleration of the tail beam can reach 0.7. Although the difference ratio of the centroid velocity of the tail beam is minimum, it can also reach 0.4. When the coal gangue separated from the shield beam and impacted on the tail beam, the impact contact response between coal and gangue is still significantly different.

### 5.2. Contact Response Difference of the Indirect Contact and Associated Part

**Figure 10:**
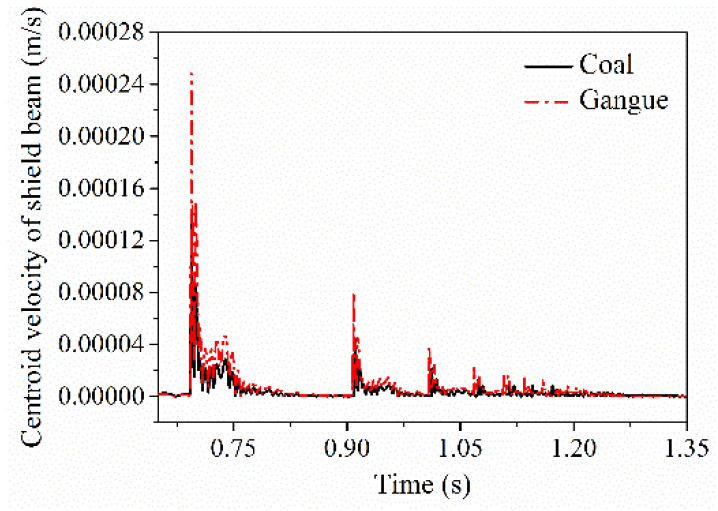
Velocity of shield beam.

**Figure 11:**
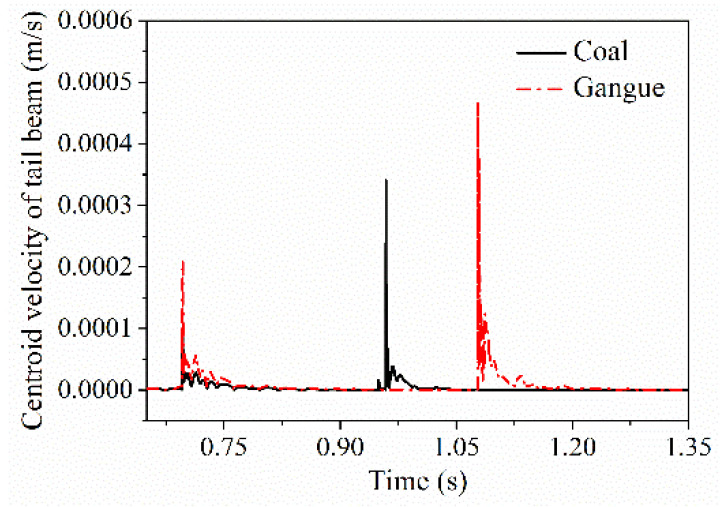
Velocity of tail beam.

**Figure 12:**
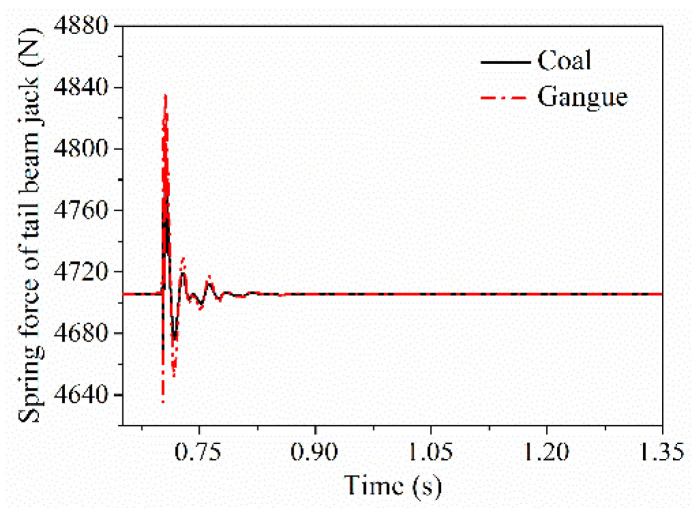
Spring force of tail beam.

Contact action between coal gangue and the hydraulic support will also be transmitted through the force transfer effect among various parts of the support, thus causing the indirect response of the related parts. In the process of impact contact between coal gangue and the different parts of the hydraulic support, three kinds of associated responses are existed, which are the vibration response of the shield beam and the tail beam jack after the tail beam is impacted by coal gangue, the associated vibration response of the top beam and the tail beam after the shield beam is impacted by coal gangue, and the vibration response of the prop and the shield beam after the top beam is impacted by coal gangue. Figure 10, Figure 11 and Figure 12 show the centroid velocity response curve of the shield beam after the tail beam is impacted by coal gangue, the centroid velocity response curve of the tail beam after the shield beam is impacted by coal gangue, and the equivalent spring vibration response curve of the tail beam jack after the tail beam is impacted by coal gangue, respectively. It can be seen from the figures that after the impact of coal gangue, there are also significant differences in the correlation responses of non-directly contacting parts. After the different parts of the hydraulic support is impacted by gangue, the contact responses of the associated parts such as the centroid velocity of the shield beam, the centroid velocity of the tail beam and the equivalent spring force amplitude of the tail beam jack are significantly greater than that of coal.

The ultimate contact response of the indirect contact and associated parts of the top beam, the shield beam and the tail beam, respectively, were extracted, as shown in Table 4. The difference ratios of the vibration contact response of the associated parts caused by the impact of coal and gangue are all greater than 0.8. The difference ratio of the centroid vibration acceleration of the associated part is much greater than the difference ratio of the centroid vibration velocity. The sensitivity of the associated parts to the contact response when the shield beam and the tail beam are impacted are both greater than that of the top beam.

The ultimate variation of the equivalent springs for the prop and the tail beam jack when impacted by coal gangue, respectively, were extracted, as shown in Table 5. The response force of each spring after coal impact is lower than that of gangue, and the difference ratio is more than 0.8, that is, there is an obvious difference in the contact response of the hydraulic system. When the top beam and the tail beam are impacted, the front and rear props and the tail beam jacks are directly stressed, respectively. The equivalent spring force, and its difference for the tail beam jack when the tail beam is impacted, is larger than the equivalent spring force and the equivalent spring force difference in the front and rear props. When three different parts were impacted, indirect contact response was observed at the tail beam jack, among which, the difference of the spring response force for the tail beam jack caused by the impacting of coal gangue was more significant when the tail beam was directly impacted.

### 5.3. Contact Response Differences of the Force Transmission Hinge Points

The top beam and the shield beam as well as the shield beam and the tail beam are connected by pin shafts. The connected positions hinge point 1 and hinge point 2, respectively, were defined, and the force transfer between the top beam, the shield beam and the tail beam is realized by hinge point 1 and hinge point 2. The ultimate variation values of the force at the two hinge points are extracted, respectively, as shown in Table 6.

When gangue impacts on the hydraulic support, the force at the hinge point is greater than that of coal, and the difference ratio is above 0.7. Compared with the contact force, the variation value of the active force at the hinge point after coal gangue impact is very small, and the active force at the hinge point 2 when coal gangue impacts on the top beam and the active force at the hinge point 1 when coal gangue impacts on the tail beam is small. By comparing the forces of hinge point 1 when coal gangue impacts on the top beam, the forces of hinge point 1 and hinge point 2 when coal gangue impacts on the shield beam, and the forces of hinge point 2 when coal gangue impacts on the tail beam, the difference in the active force for hinge point 1 when coal gangue impacts on the shield beam is the largest, while the difference in the active force for hinge point 1 when coal gangue impacts on the top beam is the smallest. Additionally, the difference ratios of the active force for two hinge points when coal gangue impacts the shield beam are both the largest.

## 6. Conclusions

In order to further clarify the contact action law and contact difference characteristics between the coal or gangue particle and the hydraulic support, and then to further lay a foundation for studying the interaction characteristics between coal gangue particles and the hydraulic support in the drawing stage of top coal caving mining, the impact contact behavior between the single coal/gangue particle and the different parts of hydraulic support is taken as the research object in this paper. Based on the construction of the accurate equivalent spring stiffness mathematical model of the hydraulic cylinder, the rigid–flexible coupling impact contact dynamic simulation model between single coal gangue particle and the hydraulic support was established by griding treatment to coal gangue particles and the main force transmission parts of the hydraulic support, and the simulation study on impact contact behavior between coal gangue and hydraulic support is carried out. The following conclusions are drawn:

(1) When the same coal particle impact on the top beam, shield beam and tail beam, respectively, from the same height of 0.8 m in the free-falling way, the coal particles collide with the top beam continuously more than 10 times, collide with the shield beam twice, then separate and eventually come into contact with the tail beam with rebound impact, and then collide with the tail beam only once and then separate. It follows that the change in impacted component will lead to the change in collision times between the particle and the hydraulic support.
(2) When the same coal particle impact on the top beam, shield beam and tail beam, respectively, from the same height of 0.8 m, in the free-falling way, the change in the impacted component will lead to the change in the contact responses value, and the relationship between the contact responses between the coal particle and the impacted component is *F*_top beam_ > *F*_shield beam_ > *F*_tail beam_, *v*_top beam_ < *v*_shield beam_ < *v*_tail beam_, *a*_top beam_ < *a*_tail beam_ < *a*_shield beam_. It can be seen that the size relationship between the contact responses produced by the same particle impacting different parts of the same support is not the same.
(3) When the coal and gangue particles with the same radius impact at the same component of the hydraulic support with the same height, all the contact responses amplitude of the direct contact component, the indirect contact associated components and the force transmission hinge points when the gangue impact are larger than that of coal.
(4) When the coal and gangue particles with the same radius impact at the top beam with the same height, the contact response difference ratios of contact force, velocity and acceleration are above 0.8, and the difference ratios of contact force and acceleration are even above 1.7. When the single particle impact at the shield beam, the contact response difference ratios of contact force, velocity and acceleration are above 1.2, and the difference ratios of contact force and acceleration are even above 1.9. When the particles impact at the tail beam, the contact response difference ratios of contact force, velocity and acceleration are above 0.8, and the difference ratios of contact force and acceleration are even above 1.3. Therefore, when coal or gangue particles with the same size impact the same part of the hydraulic support, the contact response caused by coal and gangue is obviously different.
(5) When the coal and gangue particles with the same radius impact at the same component of the hydraulic support with the same height, the associated responses of the non-directly contacting parts such as the velocity, acceleration and spring force were also significantly different, with the difference ratios above 0.8. The active forces at the hinge points when gangue impacts on the hydraulic support are greater than that of the coal, which the difference ratio is above 0.7, the differences were also significant.
(6) When the impact occurs with the hydraulic support, the direct contact response, the indirect contact associated response and the contact response of the force transmission hinge points caused by coal or gangue are all significantly different. Therefore, it is feasible to identify coal and gangue based on the impact contact response.
(7) When the coal and gangue particles with the same radius impact at the tail beam with the same height, not only the contact force, velocity and acceleration of the tail beam are obviously different, but the response force difference ratio of the tail beam jacks supporting the tail beam and the force difference ratio of the hinge point between the shield beam and the tail beam are both more than 0.8. Therefore, when conduct the coal gangue recognition technology research, the vibration responses of the tail beam with the contact responses of the tail beam jack and the connecting pin of the tail beam can be used as coal gangue recognition parameters.

The research content of this paper reveals the difference rule of contact response induced by the difference in impacted location and the impacting material properties during the contact between the coal/gangue particles and the hydraulic support, thereby providing theoretical support for coal gangue recognition in top coal caving based on the contact response difference.

## Figures and Tables

**Table 1:** Dimensions of the prop and the tail beam jack.

Oil Cylinder	Front Row Prop (mm)	Back Row Prop (mm)	Tail Beam Jack (mm)
Length of the liquid column	836.2	829.7	257.1
Thickness of the cylinder bottom	32	32	50
Inside diameter of the hydraulic cylinder	230	230	140
Diameter of the liquid column	230	230	140
Outer diameter of the hydraulic cylinder	273	273	168
Diameter of the piston rod	210	210	105
Length of the piston rod	1214.5	1214.5	595
Length of the piston rod tail	131.5	131.5	110

**Table 2:** Initial impact limit contact response.

Impact Position	Top Beam			Shield Beam			Tail Beam		
	Contact Force (N)	Velocity (m/s)	Acceleration (m/s^2^)	Contact Force (N)	Velocity (m/s)	Acceleration (m/s^2^)	Contact Force (N)	Velocity (m/s)	Acceleration (m/s^2^)
Coal	11,063.6	1.2 × 10^−4^	3.0	9114.9	3.2 × 10^−4^	11.3	6792.0	5.6 × 10^−4^	9.5
Gangue	30,020.1	2.2 × 10^−4^	8.1	25,999.5	6.9 × 10^−4^	32.3	15,773.9	1.0 × 10^−3^	22.2
Difference value	18,956.5	9.8 × 10^−4^	5.1	16,884.6	3.8 × 10^−4^	21.0	8982.0	4.4 × 10^−4^	12.7
Difference ratio	1.7	0.8×	1.7	1.9	1.2	1.9	1.3	0.8	1.3

**Table 3:** Ultimate contact response when coal gangue rebound impacting the tail beam.

Contact Response	Contact Force (N)	Velocity (m/s)	Acceleration (m/s^2^)
Coal	3656.9	3.4 × 10^−4^	5.0
Gangue	5791.9	4.7 × 10^−4^	8.3
Difference value	2135.0	1.3 × 10^−4^	3.3
Difference ratio	0.6	0.4	0.7

**Table 4:** Ultimate contact response of the associated parts.

Part	Top Beam		Shield Beam				Tail Beam	
Associated Part	Shield Beam		Top Beam		Tail Beam		Shield Beam	
	Velocity (m/s)	Acceleration (m/s^2^)	Velocity (m/s)	Acceleration (m/s^2^)	Velocity (m/s)	Acceleration (m/s^2^)	Velocity (m/s)	Acceleration (m/s^2^)
Coal	1.4 × 10^−4^	0.4	2.3 × 10^−5^	0.4	9.8 × 10^−5^	0.5	6.1 × 10^−5^	0.4
Gangue	2.5 × 10^−4^	0.9	4.7 × 10^−5^	1.2	2.1 × 10^−4^	1.3	1.1 × 10^−4^	0.9
Difference value	1.1 × 10^−4^	0.5	2.4 × 10^−5^	0.8	1.1 × 10^−4^	0.9	5.0 × 10^−5^	0.5
Difference ratio	0.8	1.2	1.0	2.0	1.1	1.8	0.8	1.3

**Table 5:** Ultimate variation of the equivalent spring force.

Direct Impact Part	Top Beam			Shield Beam		Tail Beam (N)
	Front Row Prop (N)	Back Row Prop (N)	Tail Beam (N)	Associated Tail Beam (N)	Impacting Tail Beam (N)	
Coal	61.4	62.9	15.4	7.5	15.0	72.1
Gangue	110.8	113.4	27.9	14.9	54.8	131.4
Difference value	49.4	50.6	12.5	7.4	39.8	59.3
Difference ratio	0.8	0.8	0.8	1.0	2.7	0.8

**Table 6:** Force at the hinge points.

Impact Part	Top Beam		Shield Beam		Tail Beam	
	Hinge Point 1 (N)	Hinge Point 2 (N)	Hinge Point 1 (N)	Hinge Point 2 (N)	Hinge Point 1 (N)	Hinge Point 2 (N)
Coal	109.5	43.7	631.5	190.5	42.2	293.8
Gangue	205.3	75.4	1876.3	435.6	78.1	539.0
Difference value	95.8	31.7	1244.8	245.1	35.9	245.2
Difference ratio	0.9	0.7	2.0	1.3	0.9	0.8

## Data Availability

The data used to support the findings of this study are included within the article.
